# Insights into Variability of Actinorhodopsin Genes of the LG1 Cluster in Two Different Freshwater Habitats

**DOI:** 10.1371/journal.pone.0068542

**Published:** 2013-07-02

**Authors:** Jitka Jezberová, Jan Jezbera, Martin W. Hahn

**Affiliations:** 1 Biology Centre of the ASCR, v.v.i., Institute of Hydrobiology, České Budějovice, Czech Republic; 2 Research Institute for Limnology, University of Innsbruck, Mondsee, Austria; Universidad de Salamanca, Spain

## Abstract

Actinorhodopsins (ActRs) are recently discovered proteorhodopsins present in *Actinobacteria,* enabling them to adapt to a wider spectrum of environmental conditions. Frequently, a large fraction of freshwater bacterioplankton belongs to the acI lineage of *Actinobacteria* and codes the LG1 type of ActRs. In this paper we studied the genotype variability of the LG1 ActRs. We have constructed two clone libraries originating from two environmentally different habitats located in Central Europe; the large alkaline lake Mondsee (Austria) and the small humic reservoir Jiřická (the Czech Republic). The 75 yielded clones were phylogenetically analyzed together with all ActR sequences currently available in public databases. Altogether 156 sequences were analyzed and 13 clusters of ActRs were distinguished. Newly obtained clones are distributed over all three LG1 subgroups - LG1-A, B and C. Eighty percent of the sequences belonged to the acI lineage (LG1-A ActR gene bearers) further divided into LG1-A1 and LG1-A2 subgroups. Interestingly, the two habitats markedly differed in genotype composition with no identical sequence found in both samples of clones. Moreover, Jiřická reservoir contained three so far not reported clusters, one of them LG1-C related, presenting thus completely new, so far undescribed, genotypes of *Actinobacteria* in freshwaters.

## Introduction

Members of the phylum *Actinobacteria* are abundant, cosmopolitan and extremely successful inhabitants of freshwater ecosystems [1], [2], [3]. It is now widely accepted that the most abundant freshwater actinobacterial group is usually represented by the acI lineage [3]. acI bacteria are found widespread in diverse types of freshwater environments [4], [5]. Moreover, they might be capable of both carbon fixing and rhodopsin-based phototrophy [6]. A draft genome sequence of a non-cultured member of the acI lineage has been published recently [7], however there are still pure cultures missing. It has been confirmed by the genome sequence, that the GC content of acI lineage is lower than usually found in *Actinobacteria*.

It has recently been discovered that *Actinobacteria* possess variants of rhodopsin genes, so-called actinorhodopsins (ActRs, [8]). Several recent studies performed globally highlighted the broad taxonomic and ecological distribution of microbial rhodopsin genes in diverse aquatic environments [8]–[12] suggesting they might be important in the adaptation of these microbes to life on earth’s surface. The similarity of ActRs isolated from lakes in different parts of the world suggests that these genes are dispersed globally and that they may encode important functional capabilities enabling successful competition in a wide range of freshwater environments. However, their variability in freshwater habitats in Central Europe has so far remained rather undescribed.

*Actinobacteria* from freshwater habitats comprise three phylogenetic groups of actinorhodopsin genes named LG1, LG2 and PCL1 [8]. These three clades were also reported from estuaries and hypersaline lagoons, but are almost completely absent from marine environment and cluster outside of the major proteorhodopsin clade. The LG1 group, comprising mostly freshwater sequences, can be split into three subgroups, LG1-A, B and C [13]. The genes of the LG1-A group are encoded by the acI lineage [13], [14] whereas genes of the LG1-B group of ActRs are carried by the Luna lineage of *Actinobacteria.* So far undescribed LG1-C group is represented solely be clone sequences [13] and no further information is currently available.

Previous studies on ActRs were largely focused on describing the overall diversity in all three gene clusters and studied places mainly outside Europe. As our goal was to deepen the knowledge on acI actinobacterial lineage, in this study we exclusively focused on the freshwater LG1 cluster and intended to describe the actinorhodopsin gene diversity solely within this cluster. Two new clone libraries originating from two different freshwater habitats and all actinorhodopsin sequences available in public databases were analyzed to reach this goal. More specifically, the aim of our study was to describe genetic variability of the ActR gene in two distinct freshwater habitats in Europe and to search for new ActR variants and clusters. We selected two habitats known for their high actinobacterial abundance, but unknown actinobacterial diversity, as two examples of different habitat types – a large alkaline lake and a small humic reservoir.

## Materials and Methods

No specific permissions were required for sampling of both habitats, since the habitats are not privately owned and are freely accessible.

We compared two habitats. First habitat was the Jiřická reservoir (also known as Pohořský pond; area 2.5 ha; max depth 8 m; pH 7.3) a humic dammed reservoir located in Nové Hrady area in the Czech Republic (48°36'56.88"N, 14°40'34.48"E). The second habitat was previously well described Lake Mondsee, an oligo-mesotrophic deep submontane lake in a prealpine region (area 14.2 km^2^; max depth 68 m; pH 8.5) located in the Salzkammergut area in Austria (47°49′N 13°23′E). The Jiřická reservoir (Jezbera, pers. comm.) as well as Lake Mondsee [15] are known to host abundant populations of *Actinobacteria*. Both sampling campaigns were performed in July 2011. Water samples were taken from the water surface. 500 mL of water were filtered through 0.2 µm membrane filters (Poretics) and stored at −20°C until further processed. DNA was isolated from the filters using a phenol-chlorophorm extraction protocol described in detail in [16].

CARD-FISH (Catalyzed Reporter Deposition Fluorescence in situ Hybridization) analysis to follow *Actinobacteria* abundance was performed using the protocol of [17], employing the acI specific, horseradish-peroxidase labeled probe AcI-852 [13] (5‘-AAT GCG TTA GCT GCG TCG CA-3′). Samples from both habitats were counted in triplicates and the average value ±SD is presented. Bacteria were counted using DAPI staining as detailed in [18].

LG1 group specific PCR for the ActR gene was performed according to [13]. A combination of two forward primers 5′-TAYMGNTAYGTNGAYTGG-3′ and 5′-MGNTAYATHGAYTGGYT -3′ and one reverse primer 5′-ATNGGRTANACNCCCCA-3′ targeting the LG1 ActR clade were used The clone library was constructed using pGEM-T Easy Vector System (Promega) with competent *E. coli* JM109 cells, according to the protocol supplied by the manufacturer. White colonies from the plate were transferred to a new agar plate and in parallel PCRs were performed to test for the presence of inserts by using either M13 forward and reverse universal primers or M13 forward and LG1 reverse primers (WGVYPI; [9]). Positive clones were harvested from the new agar plate after overnight incubation at 37°C into tubes with 50 µL TE buffer and stored at −20°C. The positive PCR products were sent for sequencing with M13 forward primer.

Clone sequences were carefully checked and aligned using the MEGA program, version 5 [19]. After clipping of the primer sequences, the obtained sequences had total lengths of 329–335 bp (109–111AA). The phylogenetic tree was calculated from 291 bp long sequences (98 amino acids). All available ActR sequences from the NCBI database were added to the alignment. For the phylogenetic analyses, 81 more sequences were selected apart from our 75 clone sequences; giving altogether 156 sequences. Neighbor joining method (bootstrap 1000) was conducted in Mega 5, Maximum Likelihood (best tree and bootstrap 1000) was calculated with GARLI software [20], ML consensus was performed in PAUP* [21] and Bayesian Inference (1000000 generations, 10% burnin) was calculated in MrBayes program [22]. Trees were processed in FigTree [23].

The sequences of the newly introduced clones were submitted to GenBank under accession numbers KC601681-KC601755.

## Results and Discussion

The bacterioplankton of the two sampled temperate habitats contained 24.6% ±5.3% (2.9 x 10^6^ cells per mL) and 14.8% ±3.2% (2.2×10^6^ cells per mL) of acI *Actinobacteria* in Lake Mondsee and in the Jiřická reservoir, respectively. The two established clone libraries yielded in total 75 different partial sequences of ActR genes, 38 from Jiřická and 37 from Mondsee. Regarding acI *Actinobacteria*, Lake Mondsee has been a subject of two previous, mostly isolation, studies – [15], [24] where also high abundance of *Actinobacteria* was observed. For the Jiřická reservoir, unpublished data are available, confirming high proportions of *Actinobacteria* (Jezbera pers. comm.).

**Figure 1:**
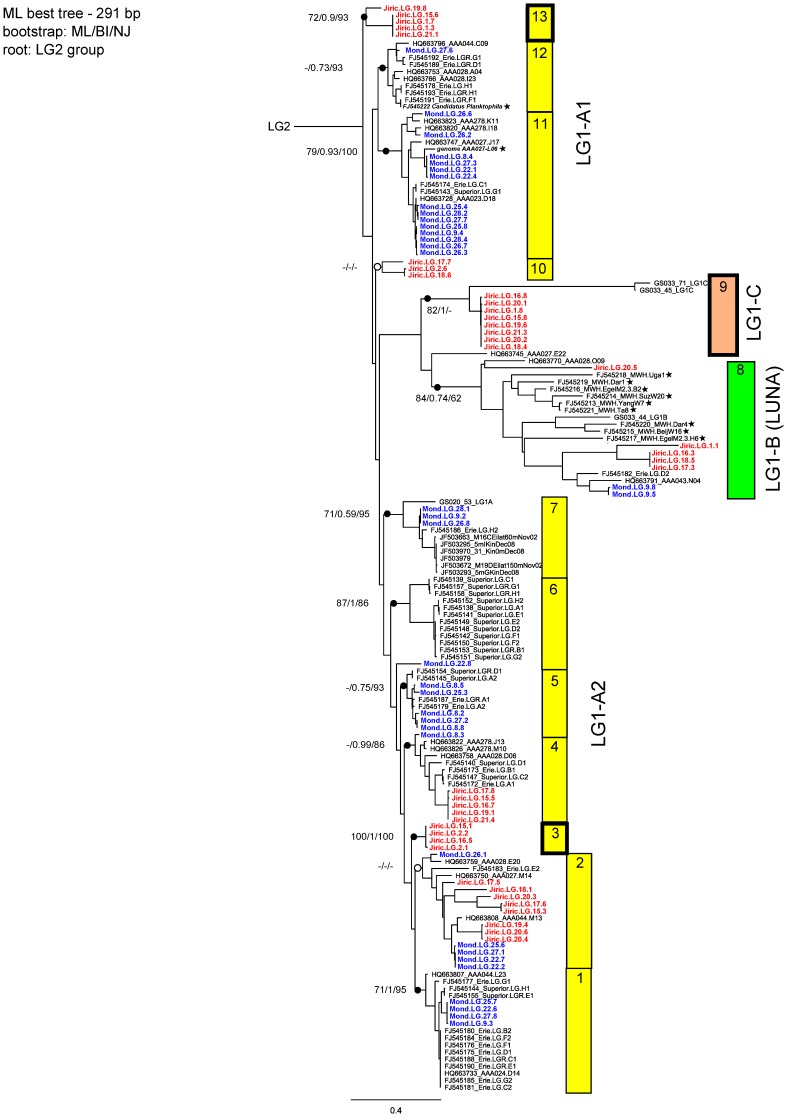
Maximum Likelihood tree of LG1 actinorhodopsin nucleotide sequences. ML best tree of LG1 actinorhodopsin nucleotide sequences (291 bp). Rooted with LG2 sequences (GS020_39, GS012_40, GS012_3). Bootstrap values from Maximum likelihood/Bayesian inference/Neighbor joining methods are depicted. Clones from Lake Mondsee are highlighted in blue and clones from Jiřická reservoir in red. Star - reference sequence (16S rRNA gene sequence is available). Numbers on the side symbolize cluster numbers, color the group affiliation (yellow = LG1-A, rosa = LG1-C, green = LG1-B). Bold frames – new clusters.

The largest proportion of our clones could be attributed to the LG1-A group, which also supports the common finding that the acI group represents the most abundant freshwater group among all *Actinobacteria* [3]. We constructed a completely new ActR phylogeny (Fig. 1) combining our newly introduced clones with all sequences currently available in GenBank, some of the metagenomic data from the Global Ocean Sampling (GOS, [9]), and included also the ActR sequence from the recently published acI genome draft [7]. The phylogenetic trees revealed 13 different gene clusters, by all three clustering methods that were mostly bootstrap confirmed. Clones obtained in this study were distributed all over the tree. This new ActRs phylogenetic tree can be divided into three main groups similar to those defined by [13]. In [13], they described that the ActR genes in freshwater habitats cluster into LG1-A (supposedly acI *Actinobacteria*), LG1-B (LUNA *Actinobacteria*) and LG1-C. Based on our results, we further divided the LG1-A group into two smaller sub-groups (LG1-A1, LG1-A2), as they markedly differ in their positions in the nucleotide tree (Fig. 1) and NA sequence similarity between these two sub-groups is only 75.8%.

**Figure 2:**
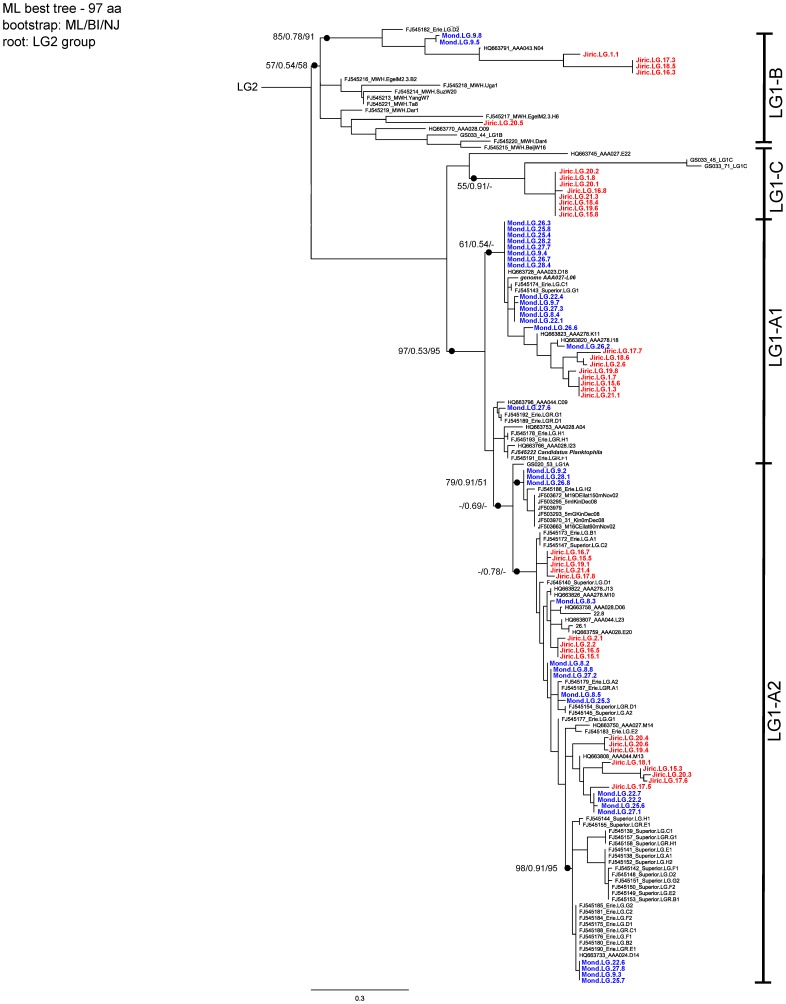
Maximum Likelihood tree of LG1 actinorhodopsin amino acid sequences. ML best tree of LG1 actinorhodopsin amino acid sequences (97 aa). Rooted with LG2 sequences (GS020_39, GS012_40, GS012_3). Bootstrap values from Maximum likelihood/Bayesian inference/Neighbor joining methods are depicted. Clones from Lake Mondsee are highlighted in blue and clones from Jiřická reservoir in red.

The sequence similarity within and among these four groups is summarized in Table 1 and Table 2, respectively. The ActR sequence obtained from the acI draft genome (genome AAA027-L06) mentioned above, clustered within the LG1-A1 group, which confirmed the previous speculations by [13] and [14] that acI *Actinobacteria* are bearers of the LG1-A actinorhodopsins. Furthermore, the ActR sequence of *Candidatus Planktophila limnetica*, a taxon representing a freshwater actinobacterium in a mixed culture [25], also clustered in the LG1-A1 group (Fig. 1). The phylogenetic position of the LG1-A1 was unstable in our performed phylogenetic analyses. The position of this cluster in the NA tree was different from its position in the AA phylogenetic tree (Fig. 2). Newly described second part of the LG1-A group, the LG1-A2 cluster, showed a stable position between the trees, but evidence linking this cluster directly to acI *Actinobacteria* via isolate or genome sequence data is missing. Despite the nucleotide sequence difference to the LG1-A reference sequences, we may assume that LG1-A2 also belongs to acI, considering mainly the AA similarity, high number of acI bacteria in the freshwaters and high number of clones in LG1-A2. Moreover, we observed high interclade diversity and seven separated clusters were observed inside of the LG1-A2 ActR gene cluster, and only one of them was not bootstrap confirmed.

**Table 1:** Amino acid sequence similarity.

LG1 group	new clones		all sequences	
A1	0.083	91.70%	0.099	90.10%
A2	0.112	88.80%	0.113	88.70%
A	0.136	86.40%	0.134	86.60%
B	0.237	76.30%	0.283	71.70%
C	0.005	99.50%	0.125	87.50%

Amino acid sequence similarity (97 aa) calculated as mean distance for the presented clones and for all 156 sequences in the analyses.

**Table 2:** Between group mean distance (%) of LG1 actinorhodopsin sequences.

	A1	A2	B
A2	75.8		
B	59	59	
C	64.5	64.4	59

Between group mean distance (%) of LG1 actinorhodopsin sequences. Sequence lengths 291 bp.

LG1-B ActRs, carried by the Luna *Actinobacteria*, is genetically a very diverse group and contains a number of reliable reference sequences, originating from cultures of *Candidatus* species and other isolates [13], [26] which are all available in GenBank. We could assign in total 9% of our clones to this group. The gene diversity among our clones was on average 76.3%, and the minimum value was 60%. They occurred in both habitats at similar and rather low levels, confirming the finding of [13] that most of the ActR diversity in freshwaters does not originate from Luna, but LG1-A (acI) *Actinobacteria*. On contrary, Luna *Actinobacteria* can be observed at high numbers in hypersaline habitats where LG1-A is completely missing.

We discovered three completely new clusters of ActR genes that have not been reported so far in the current literature (Fig.1– clusters 3, 9 and 13). The most interesting seems cluster number 9, which contains eight almost identical clones (100% AAand 99.7% NA similarity, respectively) and clusters together with the LG1-C group. This ActR genotype represents almost one quarter of our clone library from the Jiřická reservoir (21%). The substantial presence of this genotype suggests its bearer represents an important freshwater *Actinobacterium* in the Jiřická reservoir. Since they show on average only 66% similarity to cluster of sequences from the GOS metagenome originating from a hypersaline lagoon (the only LG1-C representatives currently available [13]), and taking into account their tremendous GC content difference (Table 3), it makes the assignment to the LG1-C rather debatable.

**Table 3:** GC content (%) of actinorhodopsin sequences.

	new clones			other (GOS, GenBank)		
	*average*	(min-max)	*taxa*	*average*	(min-max)	*taxa*
A1	45	(43–47)	24	47	(43–48)	15
A2	46	(44–48)	36	47	(44–53)	47
B	52	(46–60)	7	49	(40–54)	13
C	47	(46–47)	8	65	(65–66)	2

GC content (%) of actinorhodopsin sequences in various LG1 groups. Sequence lengths 291 bp.

**Figure 3:**
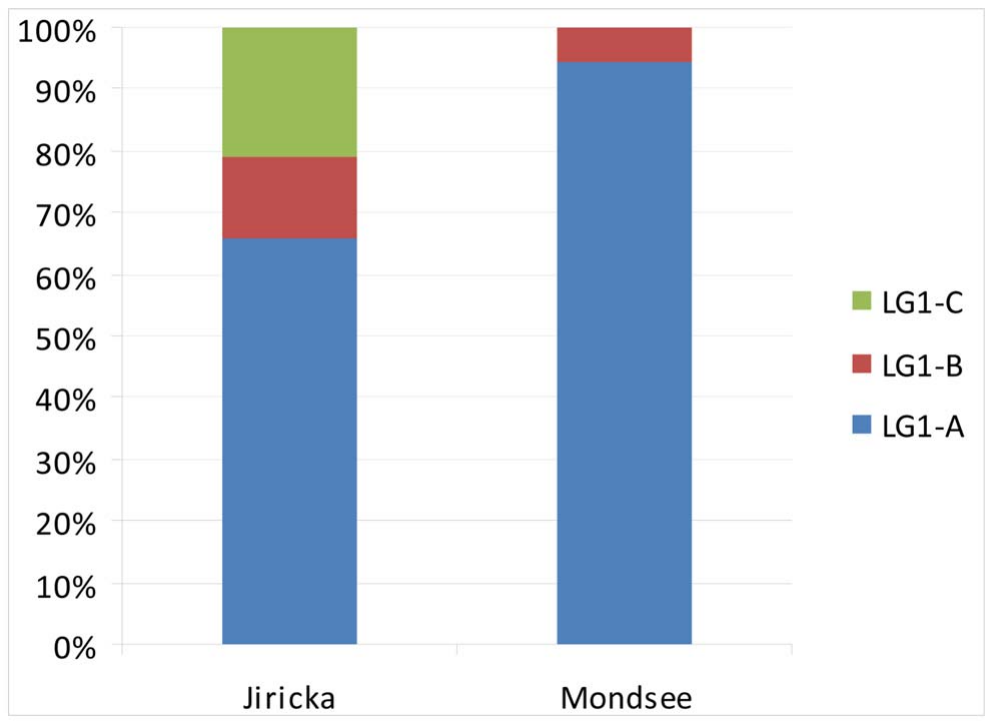
Proportions of LG1-A, B and C actinorhodopsin genotype groups. Proportions of LG1-A, B and C actinorhodopsin genotype groups in the clone libraries of Jiřická reservoir and lake Mondsee.

Both of the studied lakes differ markedly in the ActR composition (Fig. 3). Jiřická contained 66% of LG1-A, 13% LG1-B and 21% clones affiliated with cluster LG1-C. In contrast, no LG1-C genotype was observed in Mondsee. Mondsee comprised 95% of LG1-A and only 5% of LG1-B. ActR sequence similarity of all Jiřická clones was 75% (AA) and 72% (NA), and Mondsee clones 85% (AA) and 79% (NA), respectively.

**Figure 4:**
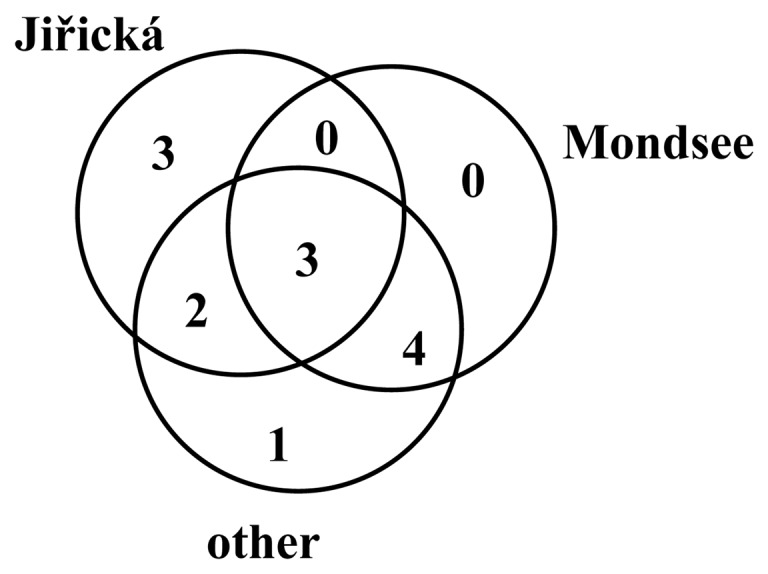
Venn diagram depicting numbers of LG1 actinorodopsin clusters. Venn diagram depicting numbers of LG1 actinorodopsin clusters defined in Fig. 1 shared by the two investigated and other habitats. For instance, Jiřická reservoir contained sequences representing three clusters not found in Lake Mondsee or one of the other habitats represented by reference sequences, while Lake Mondsee shared exclusively with Jiřická reservoir zero clusters, but four clusters with other habitats. Only three out of thirteen clusters contained sequences from all three habitat groups.

The Jiřická reservoir shared three clusters with Mondsee (Fig. 4), yet accompanied by clones from other habitats (Fig. 1– cluster 2, 4 and 8). Three clusters out of thirteen contained exclusively Jiřická clones (cluster 3, 10, 13), whereas no cluster from exclusively Lake Mondsee clones was observed. All Lake Mondsee clones were always accompanied by GenBank clones from other habitats. The fact that two studied actinobacterial communities encoded such different genotypes suggests some polemic why this may happen. (i) The two actinobacterial communities are not so different but were investigated in two completely different stages of their seasonal succession. (ii) The two habitats are colonized by completely different acI groups, which diverged a long time ago. The different actR genotypes simply reflect this long-term divergence (but the genes do not differ in their function). (iii) Differences in actR genotypes reflect different functional adaptations of the genes in these two distinct habitats.

### Conclusions

Our study evaluated overall LG1 actinorhodopsin gene variability and presented new genotypes and clusters. On two temperate freshwater habitats and sequences available from public databases we demonstrate high variability of this gene and illustrate uniqness of each place of origin. The two actinorhodopsin communities from Lake Mondsee and the Jiřická reservoir showed completely different ActR gene composition. We discovered three unique ActR gene clusters in Jiřická that were so far not reported from the literature. One of them related to the LG1-C *Actinobacteria*, presenting completely new genotype of ActR gene inherent in freshwaters. In the two studied habitats, most of the ActR diversity is formed by LG1-A (acI) Actinobacteria, not by LG1-B (Luna) Actinobacteria. Based on newly introduced sequences we suggested to further split LG1-A in two subclusters.
